# A logistic mixture model for a family-based association study

**DOI:** 10.1186/1753-6561-1-s1-s44

**Published:** 2007-12-18

**Authors:** Guan Xing, Chao Xing, Qing Lu, Robert C Elston

**Affiliations:** 1 Department of Epidemiology and Biostatistics, Case Western Reserve University, Cleveland, Ohio 44106, USA; 2 Department of Clinical Sciences, University of Texas Southwestern Medical Center, 5323 Harry Hines Boulevard, Dallas, Texas 75390-8591, USA; 3 McDermott Center for Human Growth and Development, University of Texas Southwestern Medical Center, 5323 Harry Hines Boulevard, Dallas, Texas 75390-8591, USA

## Abstract

A family-based association study design is not only able to localize causative genes more precisely than linkage analysis, but it also helps explain the genetic mechanism underlying the trait under study. Therefore, it can be used to follow up an initial linkage scan. For an association study of binary traits in general pedigrees, we propose a logistic mixture model that regresses the trait value on the genotypic values of markers under investigation and other covariates such as environmental factors. We first tested both the validity and power of the new model by simulating nuclear families inheriting a simple Mendelian trait. It is powerful when the correct disease model is specified and shows much loss of power when the dominance of a model is inversely specified, i.e., a dominant model is wrongly specified as recessive or *vice versa*. We then applied the new model to the Genetic Analysis Workshop (GAW) 15 simulation data to test the performance of the model when adjusting for covariates in the case of complex traits. Adjusting for the covariate that interacts with disease loci improves the power to detect association. The simplest version of the model only takes monogenic inheritance into account, but analysis of the GAW simulation data shows that even this simple model can be powerful for complex traits.

## Background

Linkage analysis is a useful tool for the initial exploration of complex diseases, but is limited in its ability to localize the loci potentially segregating for disease susceptibility. Association analysis, which directly tests the association between a trait and marker alleles, can more precisely localize causative genes. For this purpose a family-based association study design can be used to follow up an initial linkage scan. Moreover, it can help explain the genetic mechanism underlying the trait because extended pedigrees provide more genetic information than a random sample consisting of the same number of individuals. Note also that the often-quoted paper by Risch and Merikangas [1] for advocating an association study for detecting genes of modest effect employed a family-based association study design-in particular, using the transmission-disequilibrium test (TDT) [2] – showing it to be more powerful than a linkage analysis of affected sib pairs.

There have been several ways proposed to conduct an association study of a binary trait using general pedigrees [3,4], and there have also been joint linkage and association models proposed [5,6]. In this paper we propose a logistic mixture model for an association study of binary traits in general pedigrees. We first tested both the validity and power of this new model by simulating nuclear families inheriting a simple Mendelian trait of monogenic inheritance. We describe this initial study briefly before applying the new model to the Genetic Analysis Workshop 15 (GAW15) simulated complex trait data; in particular, we examined the performance of the model when adjusting for covariates in the case of a complex trait, as this could provide information on familial residual correlations due to common environmental sharing.

## Methods

Denote by *y*_*i *_the phenotype (*y *∈ {0, 1}, where 1 denotes affected and 0 denotes unaffected) and by *g*_*i *_the genotype of the *i*^th ^individual in a pedigree of *n *members. The likelihood for the pedigree data *Y *= (*y*_*i*_) is given by a mixture model:

$$L\left(Y\right)=\sum_{g_{1}}\sum_{g_{2}}\cdots \sum_{g_{n}}\prod_{i=1}^{n}P\left(y_{i}|g_{i}\right)P\left(g_{i}|g_{i_{F}},g_{i_{M}}\right),$$

where $P\left(g_{i}|g_{i_{F}},g_{i_{M}}\right)$ denotes the conditional probability that individual *i *has genotype *g*_*i *_given parental genotypes if he or she is a non-founder, or the probability that individual *i *has the genotype *g*_*i *_determined by population genotype frequencies if he or she is a founder; and *P*(*y*_*i*_|*g*_*i*_), the penetrance function, denotes the probability that individual *i *has phenotype *y*_*i *_given genotype *g*_*i*_. The product of these two factors is summed over all possible combinations of genotypes for the pedigree members. Assume a diallelic disease model with alleles *D *and *d*, where *D *is the disease-predisposing allele, i.e., *g*_*i *_∈ {*DD*, *Dd*, *dd*}. Define the logit of the penetrance function, log*it*[*P*(*y*_*i *_= 1|*g*_*i*_)], to be *α *+ *βg*_*i *_+ *γM*_*i *_+ *λX*_*i*_, where *M*_*i *_denotes the marker to be tested for association and *X*_*i *_denotes a vector of other covariates such as age and sex. Note that *g*_*i *_denotes the hypothetical genotype at the putative disease locus, whereas *M*_*i *_denotes the actual genotype observed for the marker to be tested for association. The constraint *P*(*y*_*i *_= 1|*DD*) = *P*(*y*_*i *_= 1|*Dd*) > *P*(*y*_*i *_= 1|*dd*) corresponds to a dominant model, *P*(*y*_*i *_= 1|*DD*) > *P*(*y*_*i *_= 1|*Dd*) = *P*(*y*_*i *_= 1|*dd*) to a recessive model, and $P\left(y_{i}=1|Dd\right)=\frac{1}{2}\left[P\left(y_{i}=1|DD\right)+P\left(y_{i}=1|dd\right)\right]$ to an additive model. The likelihood can be calculated using the Elston-Stewart [7] algorithm in the context of complex segregation analysis. The significance of a marker can be tested by comparing the likelihood with and without this marker in the model logit. Because the finite sample-size null distribution of the likelihood ratio test statistic is not known, to determine the empirical significance level of a particular observed result one can either perform a simulation study based on the null hypothesis by generating unassociated marker data for the sample at hand, or perform a permutation test (e.g., [8]). We classify the current method as model-based because a penetrance function is explicitly specified.

### Initial simulation: simple traits

We first describe our initial simulation study, for which we simulated nuclear families consisting of two parents and four children. One diallelic marker was simulated with the minor allele frequency (MAF) *p*_*D *_= 0.3, i.e., a common variant corresponding to the case for which association mapping is advocated [9], and the affection status of all individuals was simulated under nine disease models, covering the relative-risk spectrum from low to high, with the minor allele of this diallelic marker the same as the disease-predisposing allele (Table 1). Among the nine models there were three models simulated under each of dominant, recessive, and additive modes of inheritance. For example, a dominant model with penetrances (*f*_0_, *f*_1_, *f*_2_) = (0.01, 0.03, 0.03) is denoted *D*3: *D *stands for dominant mode of inheritance, 3 stands for *f*_2 _= 0.03, and logit (*f*_*i*_) = *α *+ *βg*_*i*_, where *j *denotes the number of copies of the disease allele. Under each model random families were generated and those with at least two affected children were ascertained. In this way we generated 500 replicate samples with 30 families in each sample data set. The diallelic marker and pedigree structures were simulated using the program SimPed [10]. Under the alternative hypothesis of association, the affection status of individuals was simulated according to the penetrance functions of each model; under the null hypothesis of no association, the affection status was randomly simulated according to a disease prevalence given by ${\left(1-p_{D}\right)}^{2}f_{0}+2p_{D}\left(1-p_{D}\right)f_{1}+p_{D}^{2}f_{2}$.

**Table 1:** Parameter settings for simulating disease models

Disease model^a^	*f*_0_^b^	*f*_1_^b^	*f*_2_^b^	*p*_*D*_^c^	Prevalence
*D*3	0.010	0.030	0.030	0.300	0.020
*D*6	0.010	0.060	0.060	0.300	0.036
*D*9	0.010	0.090	0.090	0.300	0.051
*R*3	0.010	0.010	0.030	0.300	0.012
*R*6	0.010	0.010	0.060	0.300	0.015
*R*9	0.010	0.010	0.090	0.300	0.017
*A*3	0.010	0.020	0.030	0.300	0.016
*A*6	0.010	0.035	0.060	0.300	0.025
*A*9	0.010	0.050	0.090	0.300	0.034

^a^*D*, *R *and *A *denote (for the minor allele) dominant, recessive, and additive modes of inheritance, respectively.

^b^*f*_*j *_denotes the penetrance of a genotype with *j *disease-predisposing alleles.

^c^Disease-predisposing allele frequency.

According to an individual's genotype, in particular the number of copies of the disease-predisposing allele, a genotypic value was assigned under the dominant, recessive, or additive model, respectively. For simplicity, we assumed Mendelian transmission probabilities and known disease allele frequencies. We analyzed each data set under three genetic models, i.e., dominant, recessive, and additive models. The significance of association between the trait and the diallelic marker was tested by fitting models with and without the diallelic marker as a covariate and then performing the likelihood-ratio test (LRT). Theoretically, this LRT statistic should asymptotically follow a $\chi_{1}^{2}$ distribution, and we report the empirical type I error rate under the null by calculating the percentage of the 500 replicate *p*-values attaining the nominal level of 0.05. Because the finite sample-size distribution of the LRT statistic is not known, we report a power determined as the percentage of the 500 replicate likelihood ratio statistics under the alternative larger than the cut-off for the top 5% of the 500 replicate LRT statistics under the null. We termed the current method SEGREG, and, as a comparison, we also analyzed the same data sets by another family-based association method [3], which is called ASSOC. The analyses were performed using the programs SEGREG and ASSOC in the Statistical Analysis for Genetic Epidemiology (S.A.G.E.) software suite, Version 5.2 [11].

## Results

### Simulation results

The two methods had very similar type I error rate and power, though SEGREG showed slightly higher power in most cases (Table 2); therefore we focus below only on the SEGREG results. The empirical type I error rate at a nominal 0.05 significance level was found to be always far below 0.05, regardless of the correctness of the model assumptions. Under the alternative, assuming a correct genetic model always results in more power than assuming a wrong model, which was anticipated. However, when analyzed under wrong assumptions, the power depends on both the underlying true model and the model assumed for the analysis. If the true disease model is dominant, analysis assuming a recessive model has little power; and if the true disease model is recessive, analysis assuming a dominant model has little power. Analysis assuming an additive model usually has fair power. However, in the case of the low penetrance disease models *D*3 and *R*3, wrongly assuming an additive model has power of only 0.530 and 0.450, respectively. Note, however, that even the power on correctly assuming an additive model is only 0.552 when the true model is *A*3. When the true disease model is additive, analysis assuming a dominant model is usually much more powerful than that assuming a recessive model.

**Table 2:** Empirical type I error rate at a nominal 0.05 significance level and power at an estimated 0.05 significance level under various assumed genetic models, based on 500 replicate samples

	Analysis model											
	
	Dominant				Recessive				Additive			
			
	SEGREG		ASSOC		SEGREG		ASSOC		SEGREG		ASSOC	
						
Simulation model	Type I error	Power	Type I error	Power	Type I error	Power	Type I error	Power	Type I error	Power	Type I error	Power
*D*3	0.006	0.828	0.006	0.818	0.014	0.074	0.010	0.080	0.000	0.530	0.000	0.524
*D*6	0.016	0.988	0.016	0.986	0.030	0.062	0.018	0.056	0.004	0.852	0.004	0.824
*D*9	0.004	1.000	0.004	1.000	0.022	0.058	0.022	0.068	0.004	0.940	0.002	0.914
*R*3	0.004	0.036	0.004	0.036	0.030	0.646	0.024	0.706	0.006	0.450	0.004	0.468
*R*6	0.008	0.030	0.010	0.028	0.020	0.998	0.022	1.000	0.002	0.978	0.004	0.982
*R*9	0.016	0.064	0.016	0.056	0.018	1.000	0.018	1.000	0.010	0.996	0.012	1.000
*A*3	0.012	0.468	0.012	0.462	0.022	0.232	0.020	0.250	0.001	0.552	0.002	0.546
*A*6	0.008	0.844	0.008	0.838	0.030	0.324	0.022	0.352	0.012	0.874	0.012	0.854
*A*9	0.010	0.944	0.014	0.936	0.016	0.550	0.010	0.574	0.001	0.962	0.004	0.960

### Application to the GAW15 simulated data: complex traits

Compared to our simulation of simple traits, the GAW15 simulation data provided an opportunity to test the new model on a complex trait. In particular, we examined the performance of the model when adjusting for covariates in the case of the data simulating rheumatoid arthritis (RA). Being aware of the answer to the simulated data that smoking interacts with locus B (MAF = 0.35) on chromosome 8 and locus F (MAF = 0.50) on chromosome 11 to increase susceptibility to RA, we performed an association analysis screening for the binary RA trait loci on chromosomes 8 and 11 using all 100 replicates of the simulated 10 K SNP data. The empirical power at a nominal 0.05 significance level for loci B and F was determined by comparing the likelihood ratio statistics to the distribution of statistics for the disease-unassociated markers SNP1_3 (MAF = 0.35) and SNP1_4 (MAF = 0.50), respectively. Age and sex were always included as covariates, and we compared the results with and without adjusting for smoking. To mimic a real situation, we only chose the first 100 nuclear families in each replicate for this study. Markers on chromosome 8 and SNP1_3 were coded with the minor allele dominant and markers on chromosome 11 and SNP1_4 were coded in an additive fashion, corresponding to the fact that loci B and F were simulated under dominant and additive models, respectively.

Without adjusting for smoking, the power of detecting loci B and F was 0.33 and 0.45 respectively, whereas the power increased to 0.35 and 0.56 after taking this covariate into consideration.

## Discussion

The new method is illustrated here on the full likelihood function of a pedigree, which is usually ascertained according to the phenotypes of probands instead of random sampling, and so, without appropriate ascertainment correction, the parameter estimates may be biased; however, this does not affect validity for testing the significance of association as a generalized linear model, though proper parameter inference can be made only when correcting for ascertainment or building the model on a conditional likelihood [12,13]. The LRT using large sample theory was very conservative for both SEGREG and ASSOC in the current study. We speculate this is because of the small sample size and we hypothesize that, as the sample size increases, the type I error rate will come closer to the nominal level. This topic awaits further investigation.

As a model-based approach, our method requires pre-specifying a genetic model and explicit penetrance functions. According to the simulation study, great power loss occurs when the dominance of a model is inversely specified, i.e., a dominant model is wrongly specified as recessive and *vice versa*, which is similar to the situation in model-based linkage analysis [14]. This is no surprise, because both are built on the full likelihood function of a pedigree. In general, the results suggest using an additive model in practice. However, in the common variant low penetrance scenario (*D*3, *R*3, *A*3), under the true genetic mechanism both dominant and recessive models have appreciably higher power than an additive model (0.828, 0.646, and 0.552, respectively). The fundamental reason for this lies in the three components of the mixture distributions not being as easily distinguishable as two components in the case of low penetrance. It is of interest to note that ASSOC, as a model-free method in the sense that no penetrance function is specified, shows similar sensitivity to the marker coding scheme as SEGREG. Because most association methods require coding SNPs in a dominant, recessive, or additive fashion, we speculate that this observation is applicable to most methods. Therefore, caution should be taken when coding markers regardless of the method employed to test association.

Detecting variants of modest effect remains a challenge for association studies, especially in the case of rare variants, which were not even simulated in the current study. For the GAW simulation data, the proposed method showed more power in detecting locus F than in detecting locus B. The effect of locus F was simulated via a variance-component method for the continuous trait IgM, which in turn affected the RA trait, whereas the effect of locus B was simulated under a dominant model with relative risk equal to 1.5. There is no direct way to compare their effect sizes. Because only locus F was fairly detected, we speculate that, if measured on the same scale, the effect size of locus B would be much more moderate.

The main limit of our method lies in its assumption of a monogenic disease mechanism without allowing for familial correlation due to polygenic and/or common environmental effects, which is unrealistic for complex diseases (though widely adopted by most methods in the literature). Model-based methods should use models that approximate the complexity of the disease being studied in order to be both robust and powerful. Analyses by models that ignore residual familial correlation can result in decreased power; how to model the familial correlations is a topic for further investigation, though we could easily incorporate covariates that resemble the action of familial correlations into the current model.

## Competing interests

The author(s) declare that they have no competing interests.
